# A high-resolution map of the gut microbiota in Atlantic salmon (*Salmo salar*): A basis for comparative gut microbial research

**DOI:** 10.1038/srep30893

**Published:** 2016-08-03

**Authors:** Karina Gajardo, Ana Rodiles, Trond M. Kortner, Åshild Krogdahl, Anne Marie Bakke, Daniel L. Merrifield, Henning Sørum

**Affiliations:** 1 Department of Basic Sciences and Aquatic Medicine, Faculty of Veterinary Medicine and Biosciences, Norwegian University of Life Sciences (NMBU), Oslo, Norway; 2 Aquaculture and Fish Nutrition Research Group, School of Biological Sciences, Plymouth University, UK; 3 Department of Food Safety and Infection Biology, Faculty of Veterinary Medicine and Biosciences, Norwegian University of Life Sciences (NMBU), Oslo, Norway

## Abstract

Gut health challenges, possibly related to alterations in gut microbiota, caused by plant ingredients in the diets, cause losses in Atlantic salmon production. To investigate the role of the microbiota for gut function and health, detailed characterization of the gut microbiota is needed. We present the first in-depth characterization of salmon gut microbiota based on high-throughput sequencing of the 16S rRNA gene’s V1-V2 region. Samples were taken from five intestinal compartments: digesta from proximal, mid and distal intestine and of mucosa from mid and distal intestine of 67.3 g salmon kept in seawater (12–14 °C) and fed a commercial diet for 4 weeks. Microbial richness and diversity differed significantly and were higher in the digesta than the mucosa. In mucosa, Proteobacteria dominated the microbiota (90%), whereas in digesta both Proteobacteria (47%) and Firmicutes (38%) showed high abundance. Future studies of diet and environmental impacts on gut microbiota should therefore differentiate between effects on mucosa and digesta in the proximal, mid and the distal intestine. A core microbiota, represented by 22 OTUs, was found in 80% of the samples. The gut microbiota of Atlantic salmon showed similarities with that of mammals.

The recent development of new analytical tools and their use for detailed study of microbiota populating body surfaces have contributed to a new understanding of the vital importance of the microbiota for the health and welfare of the host^1^. The gut plays a pivotal role, harbouring the largest microbiota populations in the body and exposing these to the body’s largest immunogenic organ, i.e. the gut wall. A rapidly increasing number of studies in man and other mammals have supplied fundamental information regarding characteristics of resident gut microbes. They are involved in modulating a variety of gut functions, including digestion and absorption of nutrients and signalling from the myriad of gut mucosal receptors to abdominal organs as well as the brain^2^,^3^. Gut dysbiosis seems to be implicated in a number of diseases, including but not limited to obesity, colitis and inflammatory bowel diseases^4^.

The microbiota of the fish gut is also receiving increased attention, illustrated by the steady increase in the development and commercial application of “functional” fish feeds for species in production. These contain e.g. pre- and/or probiotics that purportedly exert modulating effects on the gut microbiota and thereby benefit fish growth or disease resistance. However, information and understanding regarding fish gut microbiota still lags behind that of man and other mammals, including evidence for cause-effect relationships between gut microbiota and host physiology. This is at least partially due to the wide range of both external and internal factors that can influence the diversity of microorganisms described in the fish gut^3^,^5^,^6^,^7^,^8^,^9^,^10^. Specifically, environmental factors such as water temperature, salinity and geographical location, as well as developmental stage, diet, farm management practices, medical interventions and stress have all been demonstrated to extensively modulate the gut microbiota^6^,^9^,^11^,^12^,^13^,^14^,^15^,^16^. The phyla Proteobacteria, Firmicutes, Bacteroidetes, Actinobacteria and Fusobacteria have been reported as residents of the gut in a variety of fish species^17^. But the descriptions are rather incomplete regarding regional variation along the intestine^9^,^10^,^11^,^18^,^19^,^20^,^21^ and distinction between the mucosa-associated autochthonous and the more transient or digesta-associated allochthonous microbial communities^6^,^8^,^13^,^22^,^23^,^24^. Additionally, attempts to compare salmonid and mammalian gut microbiota^25^ may jump-start attempts to link our increasing knowledge of salmonid intestinal microbiota with their possible functional properties.

As one of the most important cultured fish species, the composition of the gut microbiota of Atlantic salmon has been studied to some extent, mostly by applying culture-based techniques (reviewed by Cahill^26^, and Ringø *et al*.^14^) and only recently semi-quantitative molecular techniques have been put to use^6^,^10^,^18^,^19^,^24^ and now also high-throughput sequencing^11^,^16^,^27^,^28^. These recent studies have broadened our understanding of the microbiota populations in the salmon gut, but again, a detailed regional and spatial (digesta vs mucosa-associated microbial communities) characterization, as well as comparative aspects have to our knowledge not yet been reported.

The Atlantic salmon may serve as a model species for gut microbial research for a number of reasons. From an ecological perspective, its anadromous nature is of interest and its sequenced genome greatly facilitates investigations of interactions between gut microbiota and host geno- and phenotype. In addition, salmon are strictly piscivorous in nature, and yet Norwegian farmed salmon at later growth stages are now fed diets containing more than 70% plant materials^29^. No other production animal has experienced a comparable change in diet composition. A large body of literature has described the various effects of alternative plant-based nutrient sources in fish (reviewed by Gatlin *et al*.^30^). Atlantic salmon appear particularly susceptible to gut malfunctions caused by plant antinutrients^31^,^32^ and therefore constitutes a model for studies of nutritional stress and diet-related gut disorders. Connections between shifts in microbiota caused by changes in diet composition and gut health challenges have been alluded to in some studies^10^. Recent work has shown that lactic acid producing bacteria (LAB) are present in higher abundance in salmon fed a plant-based diet compared to those fed fishmeal-based diets^10^. Also wild-caught salmon have been observed to have relatively low levels of LAB^16^. Yet any substantiated conclusions rely on investigations of resident microbiota in all intestinal compartments using high-resolution methods, as well as a functional characterization of the populations under various conditions.

The aim of the present study was therefore to conduct the first in-depth characterization of the digesta and mucosa-associated microbiota of various intestinal regions in healthy post-smolt farmed salmon. The fish were fed a current commercial diet with composition developed for the size of the experimental fish, containing 43% plant and 57% marine ingredients.

## Results

### Characteristics of the high-throughput sequence data

High-throughput sequencing of bacterial DNA resulted in a total of 2.3 million raw reads. After data quality filter processing, the number of effective reads (clean reads without Cyanobacteria and filtered at 0.005%) was 814 691. The total number of operational taxonomic units (OTUs) assigned for all of the studied compartments was 914 for those clustered at 97% sequence identity. For all samples, rarefaction curves (Supplementary Figure 1A,B) showed that samples from both the digesta and mucosa reached the saturation phase. Furthermore, the good coverage index was 0.9923 ± 0.0006 (mean ± standard error of the mean (SEM)), indicating adequate depth of sequencing.

### Gut microbiota in digesta and mucosa compartments along the intestine

**Figure 1:**
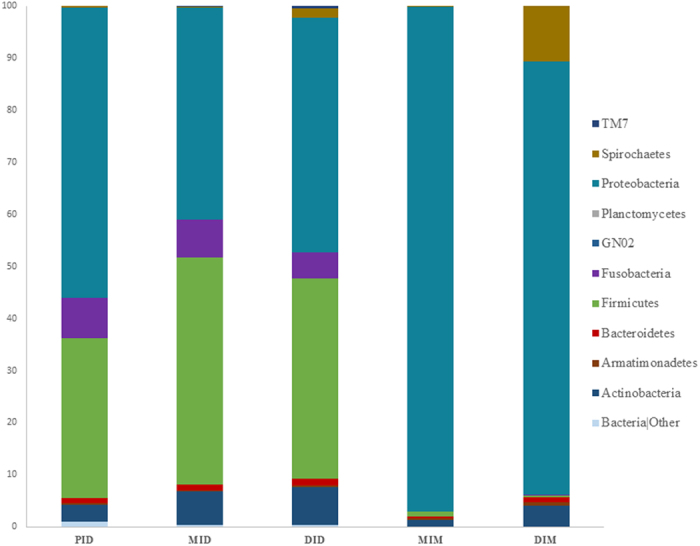
Gut microbiota composition (relative OTU composition) at phylum level. Composition of the five studied compartments: proximal intestinal digesta (PID), mid intestinal digesta (MID), distal intestinal digesta (DID), mid intestinal mucosa (MIM) and distal intestinal mucosa (DIM).

**Figure 2:**
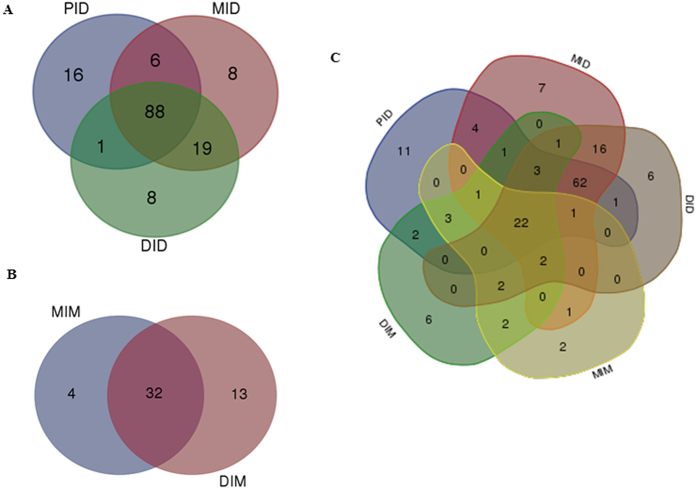
Venn diagrams showing compartmental core microbiota OTU distributions. (**A**) Digesta compartments: 88 OTUs were identified as core microbiota (80% of samples in each compartment) for the proximal intestinal digesta (PID), mid intestinal digesta (MID) and the distal intestinal digesta (DID). (**B**) Mucosa compartments: 32 OTUs were identified as core microbiota (80% of samples in each compartment) for the mid intestinal mucosa (MIM) and the distal intestinal mucosa (DIM). (**C**) Core microbiota (80% of samples in each compartment) for all studied compartments. Twenty two OTUs were found in all compartments.

Results from the analysis of the alpha diversity metrics showed significantly lower richness (Chao1 and observed species) and Shannon’s diversity index for the gut mucosa-associated microbiota compartments compared to the digesta compartments (Table 1). At phylum level, the bacterial taxonomic composition across the digesta compartments showed a high relative abundance of Proteobacteria (47%), followed by Firmicutes (38%), Fusobacteria (7%) and Actinobacteria (6%), while the mucosal compartments showed an almost complete dominance of Proteobacteria (90%; Fig. 1 and Supplementary Table 1). A total of 88 OTUs were detected as core microbiota for the digesta compartments and 32 for the mucosal compartments (Fig. 2). Across all compartments, 22 common OTUs were identified: 14 at genus level, *Janthinobacterium, Propionibacterium, Stenotrophomonas, Pseudomonas, Phyllobacterium*, *Delftia*, *Herbaspirillum*, *Burkholderia*, *Sphingomonas*, *Ochrobactrum*, *Variovorax*, *Microbacterium*, *Rhodococcus* and *Acinetobacter;* six at family level, Phyllobacteriaceae, Enterobacteriaceae, Rhizobiaceae, Comamonadaceae, Oxalobacteraceae and Caulobacteraceae; and one at order level, Rhizobiales and one at phylum level; Proteobacteria (Fig. 2 and Supplementary Table 2).

**Figure 3:**
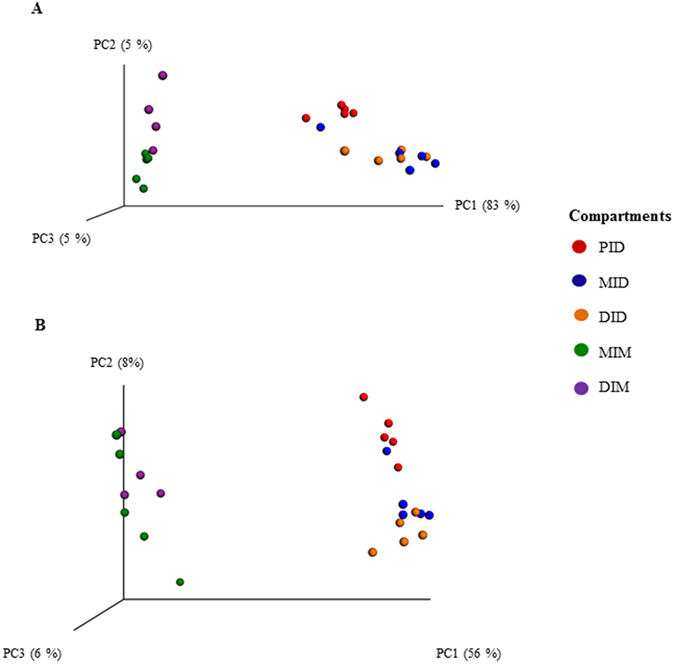
PCoA of Weighted (**A**) and Unweighted UniFrac (**B**) showing clustering of the compartments. Each dot represents one sample. Abbreviations: PID, proximal intestinal digesta; MID, mid intestinal digesta; DID, distal intestinal digesta; MIM, mid intestinal mucosa; DIM, distal intestinal mucosa.

Results from the PERMANOVA analysis revealed significant differences between most of the intestinal compartments (p = 0.001; Table 2). The exception was the difference between the mid intestinal digesta (MID) and the distal intestinal digesta (DID) (p = 0.795). Accordingly, Principal coordinates analysis (PCoA) plots (Fig. 3) based on the weighted and unweighted UniFrac metrics showed that samples within each compartment clustered together for both the digesta and the mucosa with the exception of MID and DID, which clustered together without a clear separation between them.

**Figure 4:**
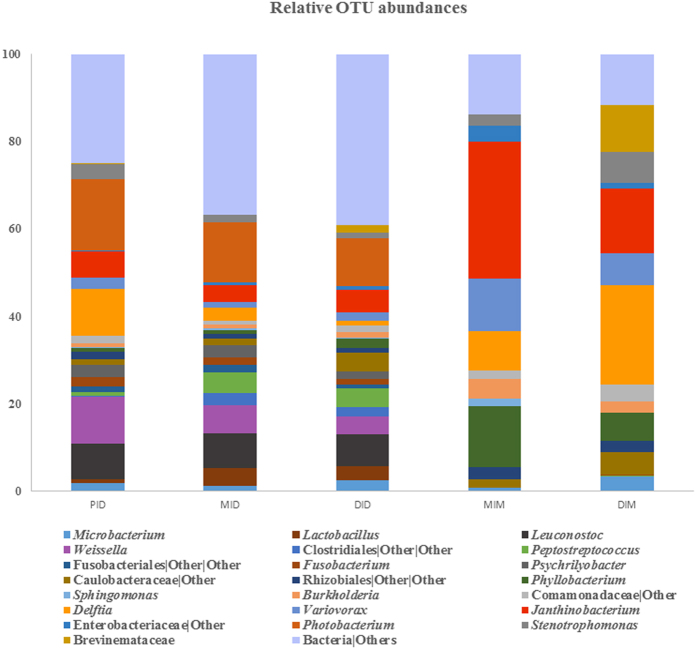
Gut microbiota composition (relative OTU composition) at genus level, or lowest taxonomic level determined by the analysis, in the gut compartments for the 12 genera showing the highest abundance. Abbreviations: PID, proximal intestine digesta; MID, mid intestinal digesta; DID, distal intestine digesta; MIM, mid intestine mucosa; DIM, distal intestine mucosa.

**Figure 5:**
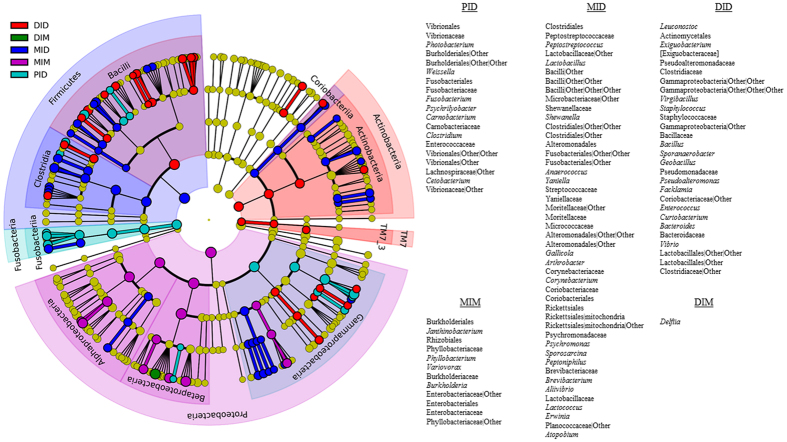
Circular cladogram reporting LEfSe results presenting the identified OTUs distributed according to phylogenetic characteristics around the circle. The dots in the centre present the OTUs at phylum level, whereas the outer circle of dots presents the OTUs at genus level. The colour of the dots and sectors indicate the compartment in which the respective OTUs are most abundant. The colour explanation is given in the upper left corner. Yellow colour indicates OTUs that showed similar abundance in all compartments. The coloured sectors give information on phylum (full name in outermost circle, given only for phylum showing significant difference between compartments), class (full name, next to the outer circle, given only for class showing significant difference between compartments). Order, family and genus that were significantly different between compartments are named at the right side of figure. Abbreviations: PID, proximal intestinal digesta (light blue); MID, mid intestinal digesta (blue); DID, distal intestinal digesta (red); MIM, mid intestinal mucosa (purple); DIM, distal intestinal mucosa (green).

Figure 4 shows the relative abundance of OTUs at the genus level in the various compartments. For the digesta, the genera *Photobacterium* (16%), *Delftia* (11%), *Weissella* (11%), *Leuconostoc* (8%), followed by *Janthinobacterium* (6%), showed the highest abundance in the proximal intestinal digesta (PID), whereas in MID and DID the genera *Photobacterium*, *Leuconostoc, Janthinobacterium, Weissella* and *Peptostreptococcus* were the most abundant. In the microbiota of the mucosa, the phylum Proteobacteria dominated and among these bacteria the genera *Janthinobacterium*, *Phyllobacterium, Variovorax* and *Delftia* showed the highest relative abundance mid intestinal mucosa (MIM), while *Delftia, Janthinobacterium*, *Variovorax* and *Stenotrophomonas* showed the highest relative abundance in the distal intestinal mucosa (DIM). In addition to genus from the Proteobacteria phylum, the family Brevinemataceae from the phylum Spirochaetes presented also high relative abundance in DIM (11%) (for a detailed list see Supplementary Table 1). In order to characterize the microbial communities that showed significant differences in abundances between the compartments, linear discriminant analysis (LDA) effect size (LEfSe) was performed. The results (Fig. 5) showed that the class Fusobacteriia and the order Vibrionales were significantly different in PID: several OTUs from the class Clostridia were significantly different in MID compared to the other compartments, whereas class Bacilli and Actinobacteria (Actinomycetales) were significantly different in DID. Compared to the digesta compartments, the mucosal compartments showed significantly higher abundance of the phylum Proteobacteria: Alphaproteobacteria and Betaproteobacteria for MIM and *Delftia* for DIM.

As the Phylogenetic investigation of communities by reconstruction of unobserved states (PICRUSt) analysis of the functional profile of the microbiota showed high Nearest sequenced taxon index (NSTI) values (0.22 ± 0.02 (mean ± SEM)) and excluded of the majority of the OTUs, the results of the predicted functional profiling were considered not to be relevant for the interpretation of functional role of the microbiota in the gut of the fish in the present investigation. The results are therefore not presented.

## Discussion

The present study provides the most detailed description of gut microbiota in Atlantic salmon to date. No studies of similar resolution regarding intestinal compartments and microbial population diversity for salmon are available in the scientific literature at present. The only other reports on gut microbiota in farmed salmon using high-throughput sequencing are from the studies of Zarkasi *et al*., one describing seasonal changes^11^ and the other the bacterial community dynamic in relation to digesta and diet properties^27^ and the study of Schmidt *et al*.^28^ describing the effect of fishmeal free diets on the microbial communities in a recirculation system. However, in those studies, only stripped faeces samples (for the first two studies) or a combination of mucosa and digesta of the mid-intestine (the last study) were investigated.

The observation that the dominating organisms in the digesta samples of our study belonged to the phyla Proteobacteria and Firmicutes is generally in agreement with previous studies of salmonids employing other methods of bacterial characterization than in the present^9^,^10^,^11^,^14^,^18^,^21^. The observed high abundance of Firmicutes, especially genera belonging to the lactic acid bacteria (LAB) *Weissella* and *Leuconostoc* also agrees with previous observations from salmonids fed diets with high content of plant ingredients^9^,^10^,^21^. However, an intermediate abundance of LAB was observed in the present study with salmon fed a diet with intermediate inclusion levels of both fishmeal and plant ingredients compared to more extreme diets containing either predominantly fishmeal or plant ingredients. In salmon fed high fishmeal diets, the abundance of LAB seems to be lower^9^,^10^. Likewise, adult wild salmon^16^ show low LAB abundance, whereas the Proteobacteria and Tenericutes dominated in their intestinal contents. Variation in abundance of these Proteobacteria and Tenericutes between the wild salmon caught at different geographical locations was not significant, strengthening the many indications of a close relationship between diet and gut microbiota. The high abundance of LAB in the cultivated salmon fed plant-rich diets is likely to be the result of the presence of carbohydrates in the diet, digestible as well as indigestible, which may preferentially be used by these bacteria as substrate for growth. Their functional significance for systemic and gut health of salmon remain to be confirmed.

In their characterization of the microbiota of the distal intestinal digesta of farmed salmon, Zarkasi *et al*.^11^ reported higher abundance of the class Gammaproteobacteria and the phyla Firmicutes and Bacteroidetes, with variation in OTU dominance between the coldest and warmest months of the year. Greater abundance was observed during the cold season for the genera *Lactococcus*, *Weissella*, *Leuconostoc*, *Cloacibacterium*, *Carnobacterium* and *Diaphorobacter*, while in the warmest months (above 16 °C) higher abundance was seen for the members of the Vibrionaceae family. Our findings from salmon held at 12 to 14 °C confirm these results, as we detected a high relative abundance of *Weissella* and *Leuconostoc* in the DID. On the other hand, and in contrast to the results of Zarkasi *et al*.^11^ the phylum Bacteroidetes and the genera *Carnobacterium* and *Lactococcus* were only found in minimal relative abundance, and, *Cloacibacterium* and *Diaphorobacter* were not detected at all in the present study. Geographical distance between Tasmania and Norway may help explain these differences between the studies. The results of Zarkasi *et al*.^11^ and those from studies of rainbow trout (*Oncorhynchus mykiss*) employing similar high-resolution tools^9^ show agreement with the current study regarding presence of *Leuconostoc*. Only a few of the earlier studies have associated *Leuconostoc* among the gut microbiota of salmon^33^,^34^,^35^, suggesting that the current approach give deeper insight into the characteristics of the gut microbiota.

Studies addressing composition of the microbiota associated with the gut mucosa in salmon are sparse^6^,^36^,^37^, and the characterization methods, intestinal sections studied and the experimental conditions have differed. However, all report a high abundance of Proteobacteria, including the present study, although the quantitative aspects differ. While the previous studies report relative abundance of Proteobacteria of 30–40%, the present study indicated a far higher abundance of more than 90% of this mucosa-associated bacteria. The cause of this seemingly major difference may be related to methodological differences, but also differences in environmental conditions including dietary differences.

In both MIM and DIM, the genus *Janthinobacterium* was highly abundant. Interestingly, the species within this genus, especially *Janthinobacterium lividum*, are known to produce a pigment known as violacein, which has antiviral, antibacterial and antifungal properties^38^. Of possible relevance are the findings indicating a mutualistic relationship between *Janthinobacterium lividum* and the red-backed salamander (*Plethodon cinereus*), preventing skin colonization and concomitant disease caused by the fungus *Batrachochytrium dendrobatidi*^38^. It is not known whether Atlantic salmon might benefit from the high abundance of *Janthinobacterium* in the gut mucosa. Its presence, metabolism and physiological effects in salmon should be addressed in future studies.

The observation of lower richness and diversity indices for the gut mucosa-associated microbiota compared to the digesta indicates that only a fraction of the bacteria present in the intestinal digesta have the characteristics necessary for colonizing the mucosa of the host. On the other hand, it is unlikely that microbes present in the mucosa are not present in the digesta. The observation in the present study of a core microbiota, represented by 22 OTU, common for all compartments is partially in agreement with results of previous studies. The presence of *Pseudomonas*, *Acinetobacter*, *Microbacterium, Janthinobacterium*, *Burkholderia*, members of the Rhizobiales and Enterobacteriaceace has been described in several studies of the salmon gut microbiota^6^,^11^,^16^,^23^,^24^,^34^. They may represent a group of well-adapted microorganisms able to colonize the gut of salmon in different environments around the world.

Our results from the PICRUSt analyses, conducted demanding clustering at 97% sequence identity, which was meant to produce a predicted functional profile, did not supply useful information. This is possibly due to qualitative and quantitative differences between our data and the databases used by PICRUSt. Similar challenges were experienced by Sullam *et al*.^39^ on samples from Trinidadian guppies. However, in the latter study, when using 94% sequence identity as criteria for OTU assignment, relevant results seemed to be produced for microbial populations. It has been suggested that results from PICRUSt should be applied with caution for new environments, especially when NSTI values are high^40^. Improvements in the existing database annotation are necessary in order to improve the accuracy and benefit from tools predicting microbial functionality in a wider range of environments^41^.

When comparing the present results regarding gut microbiota of the salmon with comparable results from terrestrial mammals, similarities were found. The most common phyla found in the salmon, and also in other fish species, i.e. Proteobacteria, Firmicutes, Actinobacteria, Fusobacteria and Bacteroidetes, together with Verrucomicrobia and Spirochaetes, are also found to be the most common phyla colonizing the gut of terrestrial mammals^17^,^42^,^43^. However, species-specific differences in the dominance of the phyla are apparent^25^. In humans and other mammals, Firmicutes and Bacteroidetes are the most dominant phyla^1^,^42^ whereas in salmon, as discussed above, the Proteobacteria and Firmicutes dominate. As in mammals, the identified microbiota in salmon differed markedly between gut regions and whether the samples originated from digesta or mucosa^42^,^44^,^45^. However, in contrast to mammals^42^ the mucosa-associated bacterial community in salmon was almost completely dominated by Proteobacteria.

## Summary and concluding remarks

The present in-depth characterization of the bacterial microorganisms in five different compartments of the gut of Atlantic salmon showed that bacterial populations varied substantially between the regions of the intestine and especially between digesta and mucosa compartments. Proteobacteria and Firmicutes were the most abundant phyla in the digesta while Proteobacteria almost completely dominated in the mucosa-associated microbiota. A core group of microbiota composed mainly of bacteria belonging to the phylum Proteobacteria was identified. Based on our data and previous reports on gut microbiota across species, we find similarities between salmon and mammalian gut microbial communities. As it is likely that the microbiota populating the mucosa interacts more closely, or at least differently, with the host than the microbiota populating the digesta, the present study strongly suggests that future work should investigate and differentiate between microbiota in digesta and mucosa when effects of diet, environment and farm management practices are investigated. This will likely facilitate achieving much needed knowledge concerning truly functional interactions between the host, diet and the microbial communities of the gut.

## Materials and Methods

### Experimental fish and environmental conditions

The experiment was conducted in a recirculation system at Nofima’s research station at Sunndalsøra, Norway in accordance with laws regulating experimentation with live animals in Norway and the experimental protocol was approved by the Norwegian Animal Research Authority (Forsøksdyrutvalget). Three groups of 40 PIT-tagged post-smolt Atlantic salmon weighing 67.3 ± 0.3 g (mean ± SEM) were kept in 500 L seawater-containing tanks with a water renewal rate of 30 L/min. Water temperature during the feeding trial varied between 12 and 14 °C, salinity between 32 and 33 g/L and oxygen saturation was above 85%. Fish were fed continuously by automatic disk feeders. A regimen of 24 h lighting was employed during the experimental period according to the routines of the facility.

### Diet and feeding

The feeding trial lasted four weeks and feed was delivered at a rate of 120% of estimated requirement in an attempt to secure *ad libitum* feed intake. A commercial, extruded diet suitable for the size and fulfilling the nutritional requirements of the fish was used, containing 45% fishmeal, 15% soy protein concentrate, 6% wheat gluten, 4% sunflower meal, 13% wheat, 12% fish oil and 5% rape seed oil. The proximate composition was 50% crude protein, 23% crude lipid, 1% crude fibre, 13% nitrogen free extract, 9% ash and 4% water. The gross energy was calculated to be 23 MJ/kg.

### Sampling procedure

At termination of the feeding period, five fish were randomly selected for sampling from the three tanks while in the fed state, two fish from two of the tanks and one from the third. Fish were anesthetized with tricaine methanesulfonate (MS222; Argent Chemical Laboratories, Redmond, WA, USA) and then euthanized by a sharp blow to the head. All sampled fish had digesta throughout the intestinal tract, ensuring intestinal exposure to the diet. The exterior of the fish was wiped clean with 70% ethanol, the abdomen opened at the ventral mid line and the whole intestine was aseptically removed from the abdominal cavity. The intestine was separated into the proximal, mid and distal intestinal regions as previously defined^46^ and each was opened longitudinally. The digesta from each of the three regions; proximal, mid and distal intestine digesta (PID, MID, DID respectively) was collected separately. The mid- and distal sections of the intestine were then washed in PBS three times to remove remnants of the digesta and samples for investigation of the mucosa-associated bacteria collected by scraping the mucosal layer from a two centimetres length of the mid-section of each region (mid intestine mucosa; MIM and distal intestine mucosa; DIM) with a sterile scalpel. Mucosal samples from the proximal intestine were not collected due to difficulties in separating the mucosa from the digesta in this region. All samples were frozen immediately in liquid N_2_, and thereafter stored at −80 °C. The PID, MID, DID, MIM and DIM are hereafter defined as separate compartments.

### DNA extraction

DNA was extracted from 100 mg of each digesta sample and 50 mg of each mucosal tissue sample. Following incubation with 50 mg mL^−1^ of lysozyme at 37 °C, DNA extraction was performed using the QIAamp Stool Mini Kit (Qiagen, Crawley, UK) according to the manufacturer’s specification with modification as described elsewhere^47^ and summarized by Falcinelli *et al*.^48^. DNA concentrations were determined using NanoDrop^TM^ 1000 spectrophotometer (Thermo Scientific, DE, USA).

### PCR amplification

To analyse the microbial populations, amplification of the variable region V1-V2 of the 16 S rRNA gene was performed. The PCR was conducted using the bacterial universal primers 27F (5′ AGA GTT TGA TCM TGG CTC AG 3′) and 338R-I (5′ GCW GCC TCC CGT AGG AGT 3′) and 338R-II (5′ GCW GCC ACC CGT AGG TGT 3′)^49^. The reaction was carried out in 50 μl sample volume using 0.4 μl of DNA template for digesta samples and 2 μl of DNA template for mucosa samples, 25 μl Phusion^®^ High-Fidelity PCR Master Mix (Thermo Scientific, CA, USA) and 1 μl of forward (27F) and reverse (pooled 338R-I and II) primers (50 pM). For digesta samples, the PCR was ran as follows: initial denaturation at 98 °C for 2 min; 30 cycles of denaturation at 98 °C for 10 s, annealing at 53 °C for 30 s, and extension at 72 °C for 60 s; followed by a final extension at 72 °C for 10 min. For mucosa samples, a touch-down PCR strategy was used and run as follows: initial denaturation at 98 °C for 3 min; 35 cycles of denaturation at 98 °C for 15 s, annealing decreasing from 63 °C to 53 °C in 10 cycles for 30 s followed by 25 cycles at 53 °C for 30 s, and extension at 72 °C for 30 s; followed by a final extension at 72 °C for 10 min. The resulting amplicons were then analysed in a 1.5% agarose gel. PCR products were purified using a QIAquick PCR Purification Kit (Qiagen, Crawley, UK) following the manufacturer’s instructions.

### High-throughput sequencing

Qubit 2.0 Fluorometer (Invitrogen, CA, USA) was used to quantify the purified PCR products. The amplicons were then evaluated for fragment concentration using Ion Library Quantitation Kit (Life Technologies, CA, USA). Concentrations were adjusted to 26 pM for all samples. Amplicons were attached to Ion Sphere Particles (ISPs) using an Ion PGM Template OT2 400 kit (Life Technologies, CA, USA) according to the manufacturer’s instructions. Multiplexed sequencing was conducted using 318 chip (Life Technologies, CA, USA) on the Ion Torrent Personal Genome platform (Life Technologies, CA, USA). Sequences were sorted by sample and filtered within the PGM software to remove low quality reads. Finally, the data from each sample was exported as individual FastQ files.

### High-throughput sequence data processing

Taxonomic analyses of sequence reads were performed after the removal of low quality scores (Q score <20 in 80% of sequences) with FASTX-Toolkit (Hannon Lab). One sample of the DIM segment presented low quality reads and therefore this sample was excluded from the analysis. The rest of the sequences were concatenated and sorted by sequence similarity into a single fasta file. Sequences were then further analysed using QIIME pipeline^50^. Greengenes database (version 13.8.) was used as reference database^51^. OTU picking was performed using the quality filter pipeline (USEARH quality filter pipeline^52^), with a 97% sequence identity. The taxonomic assignment was performed using RDP classified^53^ with a confidence of 0.8. Multiple alignment was performed with PyNAST^54^ with a minimum threshold length of 150 bp. The OTU table was made excluding the sequences that fail to align from the multiple alignment step. The resulting OTU table was filtered at 0.005% to reduce spurious OTUs^55^ and sequences classified as Cyanobacteria were removed from the final data as they were considered to be diet associated (chloroplast sequences) and do not represent populations from the gut microbiota^56^. QIIME was also used to calculate Phylogenetic tree^57^, to identify the core microbiota of the compartments, defined in this study as the operational taxonomic units (OTUs) present in 80% of the samples per compartment, and to calculate the alpha and beta diversity metrics on rarefied OTU tables. Venn diagrams representing the results of the core microbiota were draw using the web tool http://bioinformatics.psb.ugent.be/webtools/Venn/. Chao1, good coverage, Observed species and Shannon’s diversity indices were calculated. EMPeror^58^ was used to visualize the PCoA plots from the weighted and unweighted UniFrac metrics^59^. The results of the characterization of the microbiome are presented at the phylum and genus taxonomic levels.

Phylogenetic investigation of communities by reconstruction of unobserved states (PICRUSt)^40^ a tool predicting the functional profile of the microbiota was used. Since PICRUSt uses a closed reference OTU picking, based on the Greengenes database (version 13.5.), extraction of the OTUs from the original OTU table was performed using filter_otus_from_table.py script^50^. After OTUs extraction, we observed that a large number of the original 97% OTUs were excluded and only about 10% retained for PICRUSt analysis. The analysis was performed using default settings for OTU normalization by copy number, predicted gene family abundances and finally metagenome inference using KEGG orthology (KOs)^60^. Nearest sequenced taxon index (NSTI) was calculated as an estimate of the phylogenetic distance between each assigned OTU and the closest relative with a sequence reference genome.

### Statistical analysis of data

To assess the differences between the microbiota communities of the different compartments, the program PRIMER7 with PERMANOVA+^61^ was used. Permutation multivariate analysis of variance (PERMANOVA) was performed with 999 permutations to the weighted UniFrac distance matrix resulted from the beta diversity analysis of QIIME. Linear discriminant analysis (LDA) effect size (LEfSe)^62^ was used to characterize significant differences in OTUs among the compartments. The LEfSe analysis was performed using an alpha value of 0.01 for both the factorial Kruskal-Wallis rank sum test and pairwise Wilcoxon test and a threshold of 2.0 for the LDA. The approach used was an all-against-all multi-class analysis.

## Additional Information

**How to cite this article**: Gajardo, K. *et al*. A high-resolution map of the gut microbiota in Atlantic salmon (*Salmo salar*): A basis for comparative gut microbial research. *Sci. Rep.* **6**, 30893; doi: 10.1038/srep30893 (2016).

## Supplementary Material

Supplementary Information

## Figures and Tables

**Table 1:** Alpha diversity results of gut microbiota of Atlantic salmon fed a commercial diet.

	Chao1	Observed species	Shannon’s index
*Statistics*			
P (model)	0.0011	0.001	0.0004
Pooled SEM	44	41	0.3
*Means values*			
PID	552^a^	484^a^	6,4^a^
MID	629^a^	562^a^	7.4^ac^
DID	622^a^	561^a^	7.4^ac^
MIM	170^b^	132^c^	4.4^b^
DIM	188^b^	158^c^	4.9^b^

Abbreviations: PID, proximal intestine digesta; MID, mid intestinal digesta; DID, distal intestine digesta; MIM, mid intestine mucosa; DIM, distal intestine mucosa.

**Table 2:** Result of the PERMANOVA analysis of the Weighted UniFrac for the different gut compartments studied of Atlantic salmon.

	P-value
PERMANOVA	0.001
PERMANOVA Pair-wise test	
PID. MID	0.034
PID. DID	0.004
PID. MIM	0.006
PID. DIM	0.01
MID. DID	0.795
MID. MIM	0.009
MID. DIM	0.012
DID. MIM	0.009
DID. DIM	0.013
MIM. DIM	0.008

Abbreviations: PID, proximal intestine digesta; MID, mid intestinal digesta; DID, distal intestine digesta; MIM, mid intestine mucosa; DIM, distal intestine mucosa.
