# Supplementary material for: A high-resolution map of the gut microbiota in Atlantic salmon (Salmo salar): A basis for comparative gut microbial research
Source: Sci Rep. 2016 Aug 3;6:30893. doi: 10.1038/srep30893 (PMC4971465; doi:10.1038/srep30893)
Supplement: Supplementary Information [file srep30893-s1.pdf]

A high-resolution map of the gut microbiota in Atlantic salmon (Salmo salar). A basis for comparative gut microbial research

Karina Gajardo, Ana Rodiles, Trond M Kortner, Åshild Krogdahl, Anne Marie Bakke, Daniel L. Merrifield, Henning Sørum

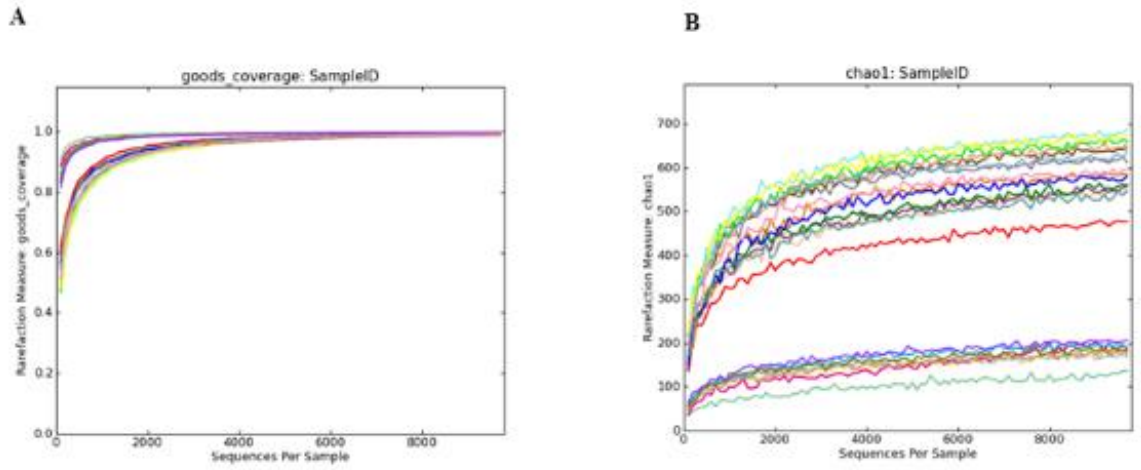

Supplementary Figure 1. Rarefaction curves for good's coverage (A) and Chao1 (B) for all analyzed samples. Each line represents one sample.

Supplementary Table 1. OTU table all segments.

Taxon	PID	MID	DID	MIM	DIM
Bacteria|Other|Other|Other|Other|Other	1	0	0	0	0
Actinobacteria|Acidimicrobiia|Acidimicrobiales|Other|Other	0	0	0	0	0
Actinobacteria|Actinobacteria|Actinomycetales|Other|Other	0	0	0	0	0
Actinobacteria|Actinobacteria|Actinomycetales|Actinomycetaceae|Actinomyces	0	0	0	0	0
Actinobacteria|Actinobacteria|Actinomycetales|Beutenbergiaceae|Salana	0	0	0	0	0
Actinobacteria|Actinobacteria|Actinomycetales|Brevibacteriaceae|Brevibacterium	0	0	0	0	0
Actinobacteria|Actinobacteria|Actinomycetales|Corynebacteriaceae|Corynebacterium	0	1	1	0	0
Actinobacteria|Actinobacteria|Actinomycetales|Dermabacteraceae|Brachybacterium	0	0	0	0	0
Actinobacteria|Actinobacteria|Actinomycetales|Dietziaceae|Other	0	0	0	0	0
Actinobacteria|Actinobacteria|Actinomycetales|Dietziaceae|Dietzia	0	0	0	0	0
Actinobacteria|Actinobacteria|Actinomycetales|Microbacteriaceae|Other	0	0	0	0	0
Actinobacteria|Actinobacteria|Actinomycetales|Microbacteriaceae|Clavibacter	0	0	0	0	0
Actinobacteria|Actinobacteria|Actinomycetales|Microbacteriaceae|Curtobacterium	0	0	0	0	0
Actinobacteria|Actinobacteria|Actinomycetales|Microbacteriaceae|Frigoribacterium	0	0	0	0	0
Actinobacteria|Actinobacteria|Actinomycetales|Microbacteriaceae|Microbacterium	2	1	3	1	3
Actinobacteria|Actinobacteria|Actinomycetales|Microbacteriaceae|Rathayibacter	0	0	0	0	0
Actinobacteria|Actinobacteria|Actinomycetales|Microbacteriaceae|Salinibacterium	0	0	0	0	0
Actinobacteria|Actinobacteria|Actinomycetales|Micrococcaceae|Other	0	0	0	0	0
Actinobacteria|Actinobacteria|Actinomycetales|Micrococcaceae|Arthrobacter	0	1	1	0	0
Actinobacteria|Actinobacteria|Actinomycetales|Micrococcaceae|Micrococcus	0	0	0	0	0
Actinobacteria|Actinobacteria|Actinomycetales|Micrococcaceae|Renibacterium	0	0	0	0	0
Actinobacteria|Actinobacteria|Actinomycetales|Nocardiaceae|Rhodococcus	0	0	0	0	1
Actinobacteria|Actinobacteria|Actinomycetales|Propionibacteriaceae|Other	0	0	0	0	0
Actinobacteria|Actinobacteria|Actinomycetales|Propionibacteriaceae|Propionibacterium	0	0	0	0	0
Actinobacteria|Actinobacteria|Actinomycetales|Sanguibacteraceae|Sanguibacter	0	0	0	0	0
Actinobacteria|Actinobacteria|Actinomycetales|Yaniellaceae|Yaniella	0	0	0	0	0
Actinobacteria|Actinobacteria|Bifidobacteriales|Bifidobacteriaceae	0	0	0	0	0
Actinobacteria|Coriobacteriia|Coriobacteriales|Coriobacteriaceae|Other	0	0	0	0	0
Actinobacteria|Coriobacteriia|Coriobacteriales|Coriobacteriaceae|Atopobium	0	0	0	0	0
Armatimonadetes|[Fimbriimonadia]|[Fimbriimonadales]|Other|Other	0	0	0	0	0
Armatimonadetes|[Fimbriimonadia]|[Fimbriimonadales]|[Fimbriimonadaceae]|Other	0	0	0	0	0
Armatimonadetes|[Fimbriimonadia]|[Fimbriimonadales]|[Fimbriimonadaceae]|Fimbriimonas	0	0	0	0	1
Bacteroidetes|Other|Other|Other|Other	0	0	0	0	0
Bacteroidetes|Bacteroidia|Bacteroidales|Other|Other	0	0	0	0	0
Bacteroidetes|Bacteroidia|Bacteroidales|Bacteroidaceae|Bacteroides	0	1	1	0	0
Bacteroidetes|Bacteroidia|Bacteroidales|Prevotellaceae|Prevotella	0	0	0	0	0
Bacteroidetes|Bacteroidia|Bacteroidales|[Paraprevotellaceae]|[Prevotella]	0	0	0	0	0
Bacteroidetes|Cytophagia|Cytophagales|Cytophagaceae|Hymenobacter	0	0	0	0	0

Bacteroidetes|Flavobacteriia|Flavobacteriales|Other|Other	0	0	0	0	0
Bacteroidetes|Flavobacteriia|Flavobacteriales|Cryomorphaceae|Fluviicola	0	0	0	0	0
Bacteroidetes|Flavobacteriia|Flavobacteriales|Flavobacteriaceae|Flavobacterium	0	0	0	0	0
Bacteroidetes|Flavobacteriia|Flavobacteriales|Flavobacteriaceae|Myroides	0	0	0	0	0
Bacteroidetes|Flavobacteriia|Flavobacteriales|Flavobacteriaceae|Polaribacter	0	0	0	0	0
Bacteroidetes|Flavobacteriia|Flavobacteriales|Flavobacteriaceae|Tenacibaculum	0	0	0	0	0
Bacteroidetes|Flavobacteriia|Flavobacteriales|Flavobacteriaceae|Zobellia	0	0	0	0	0
Bacteroidetes|Flavobacteriia|Flavobacteriales|[Weeksellaceae]|Chryseobacterium	0	0	0	0	0
Bacteroidetes|Flavobacteriia|Flavobacteriales|[Weeksellaceae]|Wautersiella	0	0	0	0	0
Bacteroidetes|Sphingobacteriia|Sphingobacteriales|Sphingobacteriaceae	0	0	0	0	0
Bacteroidetes|Sphingobacteriia|Sphingobacteriales|Sphingobacteriaceae|Pedobacter	0	0	0	0	0
Bacteroidetes|Sphingobacteriia|Sphingobacteriales|Sphingobacteriaceae|Sphingobacterium	0	0	0	0	0
Bacteroidetes|[Saprospirae]|[Saprospirales]|Chitinophagaceae|Sediminibacterium	0	0	0	0	0
Bacteroidetes|[Saprospirae]|[Saprospirales]|Saprospiraceae	0	0	0	0	0
Firmicutes|Other|Other|Other|Other	0	0	0	0	0
Firmicutes|Bacilli|Other|Other|Other	0	0	0	0	0
Firmicutes|Bacilli|Bacillales|Other|Other	0	0	0	0	0
Firmicutes|Bacilli|Bacillales	0	0	0	0	0
Firmicutes|Bacilli|Bacillales|Bacillaceae|Other	0	1	1	0	0
Firmicutes|Bacilli|Bacillales|Bacillaceae	1	1	1	0	0
Firmicutes|Bacilli|Bacillales|Bacillaceae|Anoxybacillus	0	0	0	0	0
Firmicutes|Bacilli|Bacillales|Bacillaceae|Bacillus	1	0	1	0	0
Firmicutes|Bacilli|Bacillales|Bacillaceae|Geobacillus	1	1	1	0	0
Firmicutes|Bacilli|Bacillales|Bacillaceae|Oceanobacillus	0	0	0	0	0
Firmicutes|Bacilli|Bacillales|Bacillaceae|Virgibacillus	1	1	1	0	0
Firmicutes|Bacilli|Bacillales|Listeriaceae|Brochothrix	0	0	0	0	0
Firmicutes|Bacilli|Bacillales|Paenibacillaceae|Aneurinibacillus	0	0	0	0	0
Firmicutes|Bacilli|Bacillales|Paenibacillaceae|Paenibacillus	0	0	0	0	0
Firmicutes|Bacilli|Bacillales|Planococcaceae|Other	0	0	0	0	0
Firmicutes|Bacilli|Bacillales|Planococcaceae	0	0	0	0	0
Firmicutes|Bacilli|Bacillales|Planococcaceae|Kurthia	0	0	0	0	0
Firmicutes|Bacilli|Bacillales|Planococcaceae|Lysinibacillus	0	0	0	0	0
Firmicutes|Bacilli|Bacillales|Planococcaceae|Rummeliibacillus	0	0	0	0	0
Firmicutes|Bacilli|Bacillales|Planococcaceae|Sporosarcina	0	0	0	0	0
Firmicutes|Bacilli|Bacillales|Planococcaceae|Ureibacillus	0	0	0	0	0
Firmicutes|Bacilli|Bacillales|Staphylococcaceae|Jeotgalicoccus	0	0	0	0	0
Firmicutes|Bacilli|Bacillales|Staphylococcaceae|Staphylococcus	0	1	1	0	0
Firmicutes|Bacilli|Bacillales|[Exiguobacteraceae]|Exiguobacterium	0	0	0	0	0
Firmicutes|Bacilli|Bacillales|[Thermicanaceae]|Bacillus	0	0	0	0	0
Firmicutes|Bacilli|Lactobacillales|Other|Other	0	0	0	0	0
Firmicutes|Bacilli|Lactobacillales	0	0	0	0	0

Firmicutes|Bacilli|Lactobacillales|Aerococcaceae	0	0	0	0	0
Firmicutes|Bacilli|Lactobacillales|Aerococcaceae|Aerococcus	0	0	0	0	0
Firmicutes|Bacilli|Lactobacillales|Aerococcaceae|Alkalibacterium	0	0	0	0	0
Firmicutes|Bacilli|Lactobacillales|Aerococcaceae|Facklamia	0	1	1	0	0
Firmicutes|Bacilli|Lactobacillales|Aerococcaceae|Granulicatella	0	0	0	0	0
Firmicutes|Bacilli|Lactobacillales|Carnobacteriaceae|Carnobacterium	0	0	0	0	0
Firmicutes|Bacilli|Lactobacillales|Enterococcaceae|Other	0	0	0	0	0
Firmicutes|Bacilli|Lactobacillales|Enterococcaceae|Enterococcus	0	0	0	0	0
Firmicutes|Bacilli|Lactobacillales|Enterococcaceae|Vagococcus	1	0	1	0	0
Firmicutes|Bacilli|Lactobacillales|Lactobacillaceae|Other	0	0	0	0	0
Firmicutes|Bacilli|Lactobacillales|Lactobacillaceae	0	1	0	0	0
Firmicutes|Bacilli|Lactobacillales|Lactobacillaceae|Lactobacillus	1	4	3	0	0
Firmicutes|Bacilli|Lactobacillales|Lactobacillaceae|Pediococcus	0	0	0	0	0
Firmicutes|Bacilli|Lactobacillales|Leuconostocaceae|Other	0	0	0	0	0
Firmicutes|Bacilli|Lactobacillales|Leuconostocaceae	0	0	0	0	0
Firmicutes|Bacilli|Lactobacillales|Leuconostocaceae|Leuconostoc	8	8	7	0	0
Firmicutes|Bacilli|Lactobacillales|Leuconostocaceae|Weissella	11	6	4	0	0
Firmicutes|Bacilli|Lactobacillales|Streptococcaceae|Other	0	0	0	0	0
Firmicutes|Bacilli|Lactobacillales|Streptococcaceae|Lactococcus	0	1	0	0	0
Firmicutes|Bacilli|Lactobacillales|Streptococcaceae|Streptococcus	0	1	0	0	0
Firmicutes|Clostridia|Other|Other|Other	0	0	0	0	0
Firmicutes|Clostridia|Clostridiales|Other|Other	0	3	2	0	0
Firmicutes|Clostridia|Clostridiales|Clostridiaceae|Other	0	0	0	0	0
Firmicutes|Clostridia|Clostridiales|Clostridiaceae|Clostridium	1	1	1	0	0
Firmicutes|Clostridia|Clostridiales|Eubacteriaceae|Garciella	0	0	0	0	0
Firmicutes|Clostridia|Clostridiales|Lachnospiraceae|Other	0	0	0	0	0
Firmicutes|Clostridia|Clostridiales|Lachnospiraceae	0	0	0	0	0
Firmicutes|Clostridia|Clostridiales|Peptostreptococcaceae|Other	0	0	0	0	0
Firmicutes|Clostridia|Clostridiales|Peptostreptococcaceae|Peptostreptococcus	1	5	4	0	0
Firmicutes|Clostridia|Clostridiales|Peptostreptococcaceae|[Clostridium]	0	0	0	0	0
Firmicutes|Clostridia|Clostridiales|Ruminococcaceae|Other	0	0	0	0	0
Firmicutes|Clostridia|Clostridiales|Ruminococcaceae	0	0	0	0	0
Firmicutes|Clostridia|Clostridiales|Veillonellaceae|Acidaminococcus	0	0	0	0	0
Firmicutes|Clostridia|Clostridiales|[Tissierellaceae]	0	0	0	0	0
Firmicutes|Clostridia|Clostridiales|[Tissierellaceae]|Anaerococcus	0	2	1	0	0
Firmicutes|Clostridia|Clostridiales|[Tissierellaceae]|Gallicola	0	1	1	0	0
Firmicutes|Clostridia|Clostridiales|[Tissierellaceae]|Peptoniphilus	0	1	0	0	0
Firmicutes|Clostridia|Clostridiales|[Tissierellaceae]|Sporanaerobacter	0	1	1	0	0
Firmicutes|Clostridia|Clostridiales|[Tissierellaceae]|Tepidimicrobium	0	0	0	0	0
Firmicutes|Erysipelotrichi|Erysipelotrichales|Erysipelotrichaceae|Other	0	0	0	0	0
Fusobacteria|Fusobacteriia|Fusobacteriales|Other|Other	1	2	1	0	0

Fusobacteria|Fusobacteriia|Fusobacteriales|Fusobacteriaceae|Other	1	1	0	0	0
Fusobacteria|Fusobacteriia|Fusobacteriales|Fusobacteriaceae|Cetobacterium	1	1	1	0	0
Fusobacteria|Fusobacteriia|Fusobacteriales|Fusobacteriaceae|Fusobacterium	2	2	1	0	0
Fusobacteria|Fusobacteriia|Fusobacteriales|Fusobacteriaceae|Propionigenium	0	0	0	0	0
Fusobacteria|Fusobacteriia|Fusobacteriales|Fusobacteriaceae|Psychrilyobacter	3	3	2	0	0
GN02|BD1-5	0	0	0	0	0
Planctomycetes|Planctomycetia|Planctomycetales|Planctomycetaceae|Planctomyces	0	0	0	0	0
Proteobacteria|Other|Other|Other|Other	0	0	0	0	0
Proteobacteria|Alphaproteobacteria|Other|Other|Other	0	0	0	0	0
Proteobacteria|Alphaproteobacteria|Caulobacterales|Caulobacteraceae|Other	1	2	4	2	5
Proteobacteria|Alphaproteobacteria|Caulobacterales|Caulobacteraceae	0	0	0	0	0
Proteobacteria|Alphaproteobacteria|Caulobacterales|Caulobacteraceae|Caulobacter	0	0	0	0	0
Proteobacteria|Alphaproteobacteria|Rhizobiales|Other|Other	2	1	1	3	3
Proteobacteria|Alphaproteobacteria|Rhizobiales|Bradyrhizobiaceae|Other	0	0	0	0	0
Proteobacteria|Alphaproteobacteria|Rhizobiales|Bradyrhizobiaceae|Afipia	0	0	0	0	0
Proteobacteria|Alphaproteobacteria|Rhizobiales|Bradyrhizobiaceae|Bradyrhizobium	0	0	0	1	0
Proteobacteria|Alphaproteobacteria|Rhizobiales|Brucellaceae|Ochrobactrum	0	1	1	0	1
Proteobacteria|Alphaproteobacteria|Rhizobiales|Methylobacteriaceae|Other	0	0	0	0	0
Proteobacteria|Alphaproteobacteria|Rhizobiales|Methylobacteriaceae	0	0	0	0	0
Proteobacteria|Alphaproteobacteria|Rhizobiales|Methylobacteriaceae|Methylobacterium	0	0	0	0	0
Proteobacteria|Alphaproteobacteria|Rhizobiales|Phyllobacteriaceae|Other	0	0	0	1	1
Proteobacteria|Alphaproteobacteria|Rhizobiales|Phyllobacteriaceae|Phyllobacterium	1	1	2	14	6
Proteobacteria|Alphaproteobacteria|Rhizobiales|Rhizobiaceae|Other	0	0	0	0	1
Proteobacteria|Alphaproteobacteria|Rhizobiales|Rhizobiaceae|Agrobacterium	0	0	0	1	0
Proteobacteria|Alphaproteobacteria|Rhizobiales|Rhizobiaceae|Shinella	0	0	0	0	0
Proteobacteria|Alphaproteobacteria|Rhodobacterales|Rhodobacteraceae|Other	0	0	0	0	0
Proteobacteria|Alphaproteobacteria|Rhodobacterales|Rhodobacteraceae|Octadecabacter	0	0	0	0	0
Proteobacteria|Alphaproteobacteria|Rhodospirillales|Rhodospirillaceae	0	0	0	0	0
Proteobacteria|Alphaproteobacteria|Rickettsiales|mitochondria|Other	0	1	1	0	0
Proteobacteria|Alphaproteobacteria|Sphingomonadales|Other|Other	0	0	0	0	0
Proteobacteria|Alphaproteobacteria|Sphingomonadales|Sphingomonadaceae|Other	0	0	0	0	0
Proteobacteria|Alphaproteobacteria|Sphingomonadales|Sphingomonadaceae|Blastomonas	0	0	0	0	0
Proteobacteria|Alphaproteobacteria|Sphingomonadales|Sphingomonadaceae|Novosphingobium	0	0	0	0	0
Proteobacteria|Alphaproteobacteria|Sphingomonadales|Sphingomonadaceae|Sphingomonas	0	0	0	2	0
Proteobacteria|Betaproteobacteria|Other|Other|Other	0	0	0	0	0
Proteobacteria|Betaproteobacteria|Burkholderiales|Other|Other	0	0	0	0	0
Proteobacteria|Betaproteobacteria|Burkholderiales|Alcaligenaceae	0	0	0	0	0
Proteobacteria|Betaproteobacteria|Burkholderiales|Alcaligenaceae|Achromobacter	0	0	0	0	0
Proteobacteria|Betaproteobacteria|Burkholderiales|Alcaligenaceae|Alcaligenes	0	0	0	0	0
Proteobacteria|Betaproteobacteria|Burkholderiales|Alcaligenaceae|Sutterella	0	0	0	0	0
Proteobacteria|Betaproteobacteria|Burkholderiales|Burkholderiaceae|Burkholderia	1	1	1	5	3

Proteobacteria|Betaproteobacteria|Burkholderiales|Comamonadaceae|Other	2	1	1	2	4
Proteobacteria|Betaproteobacteria|Burkholderiales|Comamonadaceae	0	0	0	1	0
Proteobacteria|Betaproteobacteria|Burkholderiales|Comamonadaceae|Acidovorax	0	0	0	0	0
Proteobacteria|Betaproteobacteria|Burkholderiales|Comamonadaceae|Comamonas	0	0	0	0	0
Proteobacteria|Betaproteobacteria|Burkholderiales|Comamonadaceae|Delftia	11	3	1	9	23
Proteobacteria|Betaproteobacteria|Burkholderiales|Comamonadaceae|Methylibium	0	0	0	0	0
Proteobacteria|Betaproteobacteria|Burkholderiales|Comamonadaceae|Pelomonas	0	0	0	0	0
Proteobacteria|Betaproteobacteria|Burkholderiales|Comamonadaceae|Rhodoferax	0	0	0	0	0
Proteobacteria|Betaproteobacteria|Burkholderiales|Comamonadaceae|Variovorax	3	1	2	12	7
Proteobacteria|Betaproteobacteria|Burkholderiales|Oxalobacteraceae|Other	0	0	0	0	0
Proteobacteria|Betaproteobacteria|Burkholderiales|Oxalobacteraceae	0	1	2	2	0
Proteobacteria|Betaproteobacteria|Burkholderiales|Oxalobacteraceae|Cupriavidus	0	0	0	0	0
Proteobacteria|Betaproteobacteria|Burkholderiales|Oxalobacteraceae|Herbaspirillum	0	0	1	2	1
Proteobacteria|Betaproteobacteria|Burkholderiales|Oxalobacteraceae|Janthinobacterium	6	4	5	31	15
Proteobacteria|Betaproteobacteria|Burkholderiales|Oxalobacteraceae|Ralstonia	0	0	0	0	0
Proteobacteria|Betaproteobacteria|Neisseriales|Neisseriaceae|Other	1	0	0	0	0
Proteobacteria|Deltaproteobacteria|Desulfovibrionales|Desulfovibrionaceae	0	0	0	0	0
Proteobacteria|Deltaproteobacteria|Myxococcales	0	0	0	0	0
Proteobacteria|Deltaproteobacteria|Myxococcales|0319-6G20	0	0	0	0	0
Proteobacteria|Deltaproteobacteria|Myxococcales|Polyangiaceae	0	0	0	0	0
Proteobacteria|Deltaproteobacteria|Thermodesulfobacteriales	0	0	0	0	0
Proteobacteria|Epsilonproteobacteria|Campylobacterales|Campylobacteraceae|Arcobacter	0	0	0	0	0
Proteobacteria|Gammaproteobacteria|Other|Other|Other	1	1	1	0	0
Proteobacteria|Gammaproteobacteria|Aeromonadales|Aeromonadaceae|Other	0	0	0	0	0
Proteobacteria|Gammaproteobacteria|Alteromonadales|Other|Other	1	1	1	0	0
Proteobacteria|Gammaproteobacteria|Alteromonadales|Moritellaceae|Other	1	1	1	0	0
Proteobacteria|Gammaproteobacteria|Alteromonadales|Psychromonadaceae|Psychromonas	0	0	0	0	0
Proteobacteria|Gammaproteobacteria|Alteromonadales|Shewanellaceae|Shewanella	0	0	0	0	0
Proteobacteria|Gammaproteobacteria|Enterobacteriales|Enterobacteriaceae|Other	0	1	1	4	1
Proteobacteria|Gammaproteobacteria|Enterobacteriales|Enterobacteriaceae|Enterobacter	0	0	0	0	0
Proteobacteria|Gammaproteobacteria|Enterobacteriales|Enterobacteriaceae|Erwinia	0	0	0	0	0
Proteobacteria|Gammaproteobacteria|Enterobacteriales|Enterobacteriaceae|Proteus	0	0	0	0	0
Proteobacteria|Gammaproteobacteria|Enterobacteriales|Enterobacteriaceae|Trabulsiella	0	0	0	0	0
Proteobacteria|Gammaproteobacteria|HTCC2188	0	0	0	0	0
Proteobacteria|Gammaproteobacteria|Legionellales	0	0	0	0	0
Proteobacteria|Gammaproteobacteria|Legionellales|Coxiellaceae	0	0	0	0	0
Proteobacteria|Gammaproteobacteria|Oceanospirillales	0	0	0	0	0
Proteobacteria|Gammaproteobacteria|Oceanospirillales|Halomonadaceae|Halomonas	0	0	0	0	0
Proteobacteria|Gammaproteobacteria|Oceanospirillales|Oceanospirillaceae|Oleispira	0	0	1	0	0
Proteobacteria|Gammaproteobacteria|Pasteurellales|Pasteurellaceae|Haemophilus	0	0	0	0	0
Proteobacteria|Gammaproteobacteria|Pseudomonadales|Moraxellaceae|Other	0	0	0	0	0

Proteobacteria|Gammaproteobacteria|Pseudomonadales|Moraxellaceae|Acinetobacter	0	1	1	1	1
Proteobacteria|Gammaproteobacteria|Pseudomonadales|Moraxellaceae|Enhydrobacter	0	0	0	0	0
Proteobacteria|Gammaproteobacteria|Pseudomonadales|Moraxellaceae|Psychrobacter	0	0	0	0	0
Proteobacteria|Gammaproteobacteria|Pseudomonadales|Pseudomonadaceae|Other	0	0	0	0	0
Proteobacteria|Gammaproteobacteria|Pseudomonadales|Pseudomonadaceae	0	0	0	0	0
Proteobacteria|Gammaproteobacteria|Pseudomonadales|Pseudomonadaceae|Pseudomonas	0	0	0	1	1
Proteobacteria|Gammaproteobacteria|Vibrionales|Other|Other	1	0	0	0	0
Proteobacteria|Gammaproteobacteria|Vibrionales|Pseudoalteromonadaceae|Pseudoalteromonas	0	0	0	0	0
Proteobacteria|Gammaproteobacteria|Vibrionales|Vibrionaceae|Other	1	0	0	0	0
Proteobacteria|Gammaproteobacteria|Vibrionales|Vibrionaceae|Aliivibrio	0	0	0	0	0
Proteobacteria|Gammaproteobacteria|Vibrionales|Vibrionaceae|Photobacterium	16	14	11	0	0
Proteobacteria|Gammaproteobacteria|Vibrionales|Vibrionaceae|Salinivibrio	0	0	0	0	0
Proteobacteria|Gammaproteobacteria|Vibrionales|Vibrionaceae|Vibrio	0	0	0	0	0
Proteobacteria|Gammaproteobacteria|Xanthomonadales|Sinobacteraceae	0	0	0	0	1
Proteobacteria|Gammaproteobacteria|Xanthomonadales|Xanthomonadaceae|Other	0	0	0	0	0
Proteobacteria|Gammaproteobacteria|Xanthomonadales|Xanthomonadaceae|Pseudoxanthomonas	0	0	0	0	0
Proteobacteria|Gammaproteobacteria|Xanthomonadales|Xanthomonadaceae|Stenotrophomonas	3	2	1	2	7
Spirochaetes|Spirochaetes|Sphaerochaetales|Sphaerochaetaceae|wall-less	0	0	0	0	0
Spirochaetes|[Brevinematae]|[Brevinematales]|Brevinemataceae	0	0	2	0	11
TM7|TM7-3|EW055	0	0	0	0	0
TM7|TM7-3|I025|Rs-045	0	0	0	0	0

Supplementary Table 2. Core microbiota (OTUs present in 80% of the samples per compartment) for intestinal digesta, intestinal mucosa and for all compartments.

**Int. digesta**		**Int. Mucosa**		**All compartments**	
Phylum	OTU	Phylum	OTU	Phylum	OTU
Proteobacteria	*Janthinobacterium*	Actinobacteria	*Microbacterium*	Proteobacteria	*Janthinobacterium*
Proteobacteria	*Delftia*	Proteobacteria	*Oxalobacteraceae*	Actinobacteria	*Propionibacterium*
Proteobacteria	*Stenotrophomonas*	Proteobacteria	*Ochrobactrum*	Proteobacteria	*Stenotrophomonas*
Firmicutes	*Enterococcus*	Proteobacteria	*Sphingomonas*	Proteobacteria	*Delftia*
Firmicutes	*Weissella*	Proteobacteria	*Herbaspirillum*	Proteobacteria	*Pseudomonas*
Proteobacteria	*Oleispira*	Proteobacteria	*Burkholderia*	Proteobacteria	*Herbaspirillum*
Firmicutes	*Clostridium*	Proteobacteria	*Variovorax*	Proteobacteria	*Burkholderia*
Firmicutes	*Lactobacillus*	Proteobacteria	*Pseudomonas*	Proteobacteria	*Phyllobacterium*
Firmicutes	*Leuconostoc*	Proteobacteria	*Bradyrhizobium*	Proteobacteria	*Acinetobacter*
Firmicutes	*Staphylococcus*	Actinobacteria	*Rhodococcus*	Proteobacteria	*Sphingomonas*
Firmicutes	*Streptococcus*	Proteobacteria	*Janthinobacterium*	Proteobacteria	*Ochrobactrum*
Proteobacteria	*Pseudomonas*	Armatimonadetes	*Fimbriimonas*	Proteobacteria	*Variovorax*
Actinobacteria	*Propionibacterium*	Proteobacteria	*Acinetobacter*	Actinobacteria	*Microbacterium*
Proteobacteria	*Herbaspirillum*	Proteobacteria	*Acidovorax*	Actinobacteria	*Rhodococcus*
Proteobacteria	*Burkholderia*	Proteobacteria	*Enterobacter*	Proteobacteria	Phyllobacteriaceae
Proteobacteria	*Acinetobacter*	Actinobacteria	*Propionibacterium*	Proteobacteria	Enterobacteriaceae
Proteobacteria	*Phyllobacterium*	Proteobacteria	*Phyllobacterium*	Proteobacteria	Rhizobiaceae
Proteobacteria	*Sphingomonas*	Proteobacteria	*Stenotrophomonas*	Proteobacteria	Comamonadaceae
Proteobacteria	*Variovorax*	Proteobacteria	*Delftia*	Proteobacteria	Oxalobacteraceae
Actinobacteria	*Rhodococcus*	Proteobacteria	Sinobacteraceae	Proteobacteria	Caulobacteraceae
Fusobacteria	*Cetobacterium*	Proteobacteria	Caulobacteraceae	Proteobacteria	Rhizobiales
Proteobacteria	*Proteus*	Proteobacteria	Phyllobacteriaceae		Proteobacteria
Proteobacteria	*Vibrio*	Proteobacteria	Enterobacteriaceae		
Proteobacteria	*Erwinia*	Proteobacteria	Comamonadaceae		

Firmicutes	*Facklamia*	Proteobacteria	Rhizobiaceae		
Firmicutes	*Sporanaerobacter*	Spirochaetes	Brevinemataceae		
Actinobacteria	*Arthrobacter*	Proteobacteria	Xanthomonadaceae		
Actinobacteria	*Renibacterium*	Proteobacteria	Rhizobiales		
Fusobacteria	*Psychrilyobacter*	Proteobacteria	Legionellales		
Proteobacteria	*Ochrobactrum*	Proteobacteria	Burkholderiales		
Fusobacteria	*Fusobacterium*	Proteobacteria	Betaproteobacteria		
Actinobacteria	*Corynebacterium*		Proteobacteria		
Firmicutes	*Geobacillus*				
Actinobacteria	*Atopobium*				
Firmicutes	*Virgibacillus*				
Firmicutes	*Ureibacillus*				
Firmicutes	*Exiguobacterium*				
Firmicutes	*Acidaminococcus*				
Firmicutes	*Lactococcus*				
Firmicutes	*Sporosarcina*				
Firmicutes	*Vagococcus*				
Firmicutes	*Anaerococcus*				
Firmicutes	*Bacillus*				
Firmicutes	*Tepidimicrobium*				
Firmicutes	*Peptostreptococcus*				
Firmicutes	*Paenibacillus*				
Firmicutes	*Oceanobacillus*				
Proteobacteria	*Pseudoalteromonas*				
Firmicutes	*Anoxybacillus*				
Proteobacteria	*Psychrobacter*				
Proteobacteria	*Shewanella*				
Proteobacteria	*Photobacterium*				
Actinobacteria	*Microbacterium*				
Proteobacteria	*Aliivibrio*				

Bacteroidetes	*Bacteroides*				
Fusobacteria	Fusobacteriaceae				
Firmicutes	Bacillaceae				
Proteobacteria	Alcaligenaceae				
Proteobacteria	Pseudomonadaceae				
Firmicutes	Lachnospiraceae				
Proteobacteria	Comamonadaceae				
Firmicutes	Ruminococcaceae				
Actinobacteria	Coriobacteriaceae				
Firmicutes	Planococcaceae				
Firmicutes	Clostridiaceae				
Proteobacteria	Rhizobiaceae				
Firmicutes	Enterococcaceae				
Proteobacteria	Phyllobacteriaceae				
Proteobacteria	Enterobacteriaceae				
Actinobacteria	Bifidobacteriaceae				
Firmicutes	Lactobacillaceae				
Proteobacteria	Vibrionaceae				
Firmicutes	Leuconostocaceae				
Proteobacteria	Moritellaceae				
Proteobacteria	Oxalobacteraceae				
Proteobacteria	Caulobacteraceae				
Firmicutes	Bacillales				
Proteobacteria	Alteromonadales				
Proteobacteria	Rickettsiales				
Proteobacteria	Vibrionales				
Firmicutes	Clostridiales				
Proteobacteria	Rhizobiales				
Firmicutes	Lactobacillales				
Fusobacteria	Fusobacteriales				

Actinobacteria	Actinomycetales				
Firmicutes	Bacilli				
Proteobacteria	Gammaproteobacteria				
	Proteobacteria				
